# Single step transformation of sulphur to Li_2_S_2_/Li_2_S in Li-S batteries

**DOI:** 10.1038/srep12146

**Published:** 2015-07-15

**Authors:** M. Helen, M. Anji Reddy, Thomas Diemant, Ute Golla-Schindler, R. Jürgen Behm, Ute Kaiser, Maximilian Fichtner

**Affiliations:** 1 Helmholtz Institute Ulm (HIU), D-89081 Ulm, Germany; 2 Institute of Surface Chemistry and Catalysis, Ulm University, D-89081 Ulm, Germany; 3 Electron Microscopy Group of Materials Science, Central Facility for Electron Microscopy, Ulm University, D-89081 Ulm, Germany; 4 Institute of Nanotechnology, Karlsruhe Institute of Technology, P.O. Box 3640, D-76021 Karlsruhe, Germany

## Abstract

Lithium-sulphur batteries have generated tremendous research interest due to their high theoretical energy density and potential cost-effectiveness. The commercial realization of Li-S batteries is still hampered by reduced cycle life associated with the formation of electrolyte soluble higher-order polysulphide (Li_2_S_x_, x = 4–8) intermediates, leading to capacity fading, self-discharge, and a multistep voltage profile. Herein, we have realized a practical approach towards a direct transformation of sulphur to Li_2_S_2_/Li_2_S in lithium-sulphur batteries by alteration of the reaction pathway. A coconut shell derived ultramicroporous carbon-sulphur composite cathode has been used as reaction directing template for the sulphur. The lithiation/delithiation and capacity fading mechanism of microporous carbon confined sulphur composite was revealed by analyzing the subsurface using X-ray photoelectron spectroscopy. No higher-order polysulphides were detected in the electrolyte, on the surface, and in the subsurface of the cathode composite. The altered reaction pathway is reflected by a single-step profile in the discharge/charge of a lithium-sulphur cell.

Rechargeable lithium-sulphur (Li-S) batteries are among the most promising candidates for high energy storage devices^1^,^2^. They have gained significant interest owing to their high theoretical specific capacity (1675 mAh g^−1^) and high theoretical energy density (2600 Wh kg^−1^)^3^,^4^,^5^. In addition, sulphur is naturally abundant, inexpensive and nontoxic. Despite these advantages Li-S batteries have not been commercially realized. Major drawbacks are the insulating nature of orthorhombic sulphur (5 × 10^−30 ^S cm^−1^ at 25 °C)^6^ and its discharge product (Li_2_S)^7^, the dissolution of lithium polysulphide formed during the electrochemical process in organic liquid electrolyte^8^,^9^,^10^, and the large volumetric expansion of sulphur (80%) during lithiation. These factors result in a low utilization of active material and poor cycle life of Li-S batteries^11^,^12^,^13^,^14^.

The electrochemical discharge mechanism of sulphur cathode in Li-S battery proceeds in a stepwise^9^,^10^,^15^,^16^ manner. In the first stage of discharge, sulphur existing as cyclooctasulphur (cyclo-S_8_) is reduced to Li_2_S_4_, with a first plateau appearing at ~2.3 V vs Li^+^/Li. Various intermediate polysulphides (Li_2_S_x_, x = 8–4) formed at this stage are highly soluble in the organic electrolyte, contributing to an irreversible capacity loss. Furthermore, the dissolved higher order polysulphides diffuse to the lithium anode, get converted to lower order polysulphides (Li_2_S or Li_2_S_2_) and diffuse back to the sulphur cathode where they can reconvert to higher order polysulphides. During this shuttle insoluble polysulphides (Li_2_S or Li_2_S_2_) accumulate to form an insulating layer on the lithium anode thereby reducing the performance of the cell^17^,^18^. In the second stage of the discharge, a transformation from Li_2_S_4_ to Li_2_S_2_ occurs (plateau appearing at ~2 V vs Li^+^/Li), followed by the transformation of Li_2_S_2_ to Li_2_S^9^,^10^.

Considerable efforts have been made to overcome these problems, for example 1) by forming sulphur composites with a conductive matrix like carbon nano tubes (CNT)^19^,^20^, conductive polymer^21^, various porous^4^,^22^,^23^,^24^,^25^,^26^,^27^ and active carbons^28^, to enhance the electronic conductivity of sulphur and to confine polysulphides, 2) by modifying the electrolyte compositions^29^ or by opting to ionic liquid electrolytes^30^,^31^,^32^, polymer electrolytes or solid-state electrolytes^33^,^34^ and 3) by modifying the cell configuration^35^,^36^,^37^,^38^,^39^ to restrain polysulphide dissolution. Ultramicroporous carbons (≤0.7 nm) as sulphur hosts have gained interest due to the inspiring performance in the presence of carbonate-based electrolytes^25^,^40^ which are otherwise considered unsuitable due to the reaction of dissolved polysulphide anions with the carbonate solvent, resulting in a rapid capacity loss^29^. It was hypothesized that such carbon confines smaller allotropes of sulphur, which potentially can bypass the formation of soluble polysulphides. Nevertheless, no evidence was reported to support this hypothesis. Here, we demonstrate and clarify the direct transformation of sulphur to Li_2_S_2_/Li_2_S in Li-S batteries using a coconut shell derived ultramicroporous carbon-sulphur composite (CSC-S) cathode and a conventional carbonate-based electrolyte (1 M LiPF_6_ in 1:1 ethylene carbonate (EC)/dimethyl carbonate (DMC) (LP30, BASF). The Li-S cell with CSC-S cathode exhibited sustainable cycling performance of over 400 cycles with a Coulombic efficiency of 99.6%.

**Physical characterization of carbon and carbon-sulphur composite**

In recent work, microporous carbon utilized for Li-S batteries was prepared by employing a sacrificial template and by forming composites with CNTs^41^. As such method is complicated and costly a biomass derived carbon would be advantageous with respect to cost and availability. Among the various biomass sources for carbon that are known, coconut shell derived carbon has been reported to reproducibly produce micropores (<2 nm) under certain activation conditions^42^. Using coconut shell as an inexpensive raw material we could successfully prepare microporous carbon (CSC, represents coconut shell derived carbon), predominantly with ultra micropore (0.5 nm), which was employed as a host for sulphur. The CSC-S composite was prepared by a melt-infusion method under vacuum. A 1:1 weight ratio of coconut shell derived activated carbon (CSC) and sulphur were mixed and heated at 155 ± 1 °C for 12 h and cooled to RT under vacuum using a glass oven.

The X-ray diffraction (XRD) pattern for the CSC-S composite is compared to its respective pure components, sulphur and carbon (CSC), in Fig. 1a. The absence of peaks corresponding to sulphur in the composite indicates the lack of long range order due to the confinement of sulphur into the carbon pores. The nitrogen adsorption/desorption isotherms of CSC and CSC-S composite exhibit type I and type II isotherms, respectively. The pore size distribution curve as derived by density functional theory (DFT) calculations^43^,^44^ of carbon shows exclusively micropores, most of them with a diameter of 0.53 nm. Upon melt infusing sulphur in the carbon, a substantial decrease in Brunauer Emmett Teller (BET) surface area (from 1600 to 16 m^2^ g^−1^), and pore volume (from 0.66 to <0.02 cm^3^ g^−1^) was noticed. This is associated with a sharp decrease in the differential pore volume at pore size below 1.2 nm (0.53) (inset of Fig. 1b). From TGA (Fig. 1c), the sulphur loading in the composite was estimated to be 45.8 wt %. A weight loss occurred between 200 to 500 °C which is far above the sublimation temperature (113 °C) of elemental sulphur^45^. The complete evaporation of sulphur from the composite occurred at 500 °C, which is again above the evaporation temperature of elemental sulphur (444.6 °C^45^). This suggests a binding interaction of sulphur at the interface of the carbon micropores and the need for extra energy to remove sulphur confined inside the pores^22^,^19^,^40^. Figure 1d shows the scanning electron microscope (SEM) images of CSC and CSC-S composite compared at lower and higher magnifications. The composite exhibited flake-like morphology with irregular shapes. No segregation of sulphur on the carbon surface was observed.

The transmission electron microscopy (TEM) image of the as-synthesized CSC (Fig. 2a) reveals a disordered structure with wormhole-like micropores. A similar morphology is observed for CSC-S composite (Fig. 2c). Additional EFTEM (Energy-filtered TEM) analysis did not show an indication that the sulphur segregates on the surface. In order to elucidate the structural and electronic property of the composite electron energy loss spectroscopy (EELS) was performed. Figs S2a and S2b show the original EELS intensities of the as-synthesized CSC and CSC-S composite, respectively. The power-law background subtracted EELS spectra for the samples are shown in Fig. 2b (as-synthesized CSC), 2d and 2e (CSC-S composite). The C-K edge spectra of both samples represent two main features i) a sharp peak at 285 eV due to the 1s → π* transition and ii) a broad feature >292 eV corresponding to the 1 s → σ* transition. An increase in sharpness of the π* and σ* peak at the C-K edge is observed for CSC-S composite (Fig. 2d) in comparison to the as-synthesized CSC (Fig. 2b). The π* peak increment represents an increase in sp^2^ hybridization (graphitization) after sulphur incorporation and the σ* peak increment indicates an additional contribution originating from C-S bonds. The presence of a peak at 165.7 eV due to the S-L_2,3_ edge and an another peak at 228.7 eV due to the S-L_1_ edge confirms the existence of sulphur in the CSC-S composite (Fig. 2b).

**Electrochemical performance**

Figure 3a shows the first 10 discharge/charge voltage profiles of the CSC-S composite cathode cycled at C/20 (84 mA g^−1^). In the first cycle two discharge plateaus and one charge plateau were observed. During the first discharge the very short plateau at 2.38 V (vs Li^+^/Li) corresponds to the reaction of lithium with the carbon host in the composite electrode. Supporting this, Fig. S3 shows the corresponding discharge voltage profile for the as-synthesized carbon host (CSC). The long discharge plateau at 1.8 V (vs Li^+^/Li, from differential capacity plots (DCP) Fig. 3b) during the first cycle corresponds to the formation of lower order lithium polysulphides (Li_2_S_2_ and/or Li_2_S), it is in accordance to the lithiation potential of micropore confined sulphur reported earlier^25^,^41^. Ultramicroporous carbons are assumed to possess small sulphur allotropes (S_4_, S_3_ and S_2_) due to their spatial constrains and are assumed to form lower order polysulphides during discharge. During the first charge, a single plateau at 2.2 V (vs Li^+^/Li) corresponding to the oxidation of lower order polysulphides was observed. The short discharge plateau at 2.38 V (vs Li^+^/Li) disappeared after the first cycle. Further cycling resulted in a single discharge and charge plateau at 1.8 V and 2.2 V (vs Li^+^/Li, from DCP Fig. 3b), respectively. This is different from the two-plateau discharge (2.3 V and 2.1 V vs Li^+^/Li) and charge (2.35 V and 2.45 V vs Li^+^/Li) behavior of typical cyclo-S_8_^23^,^41^,^46^,^47^. The CSC-S cell exhibits a comparably large voltage hysteresis of 0.4 V between discharge and charge, which might be due to the reduced contact between the sulphur and electrolyte. In Fig. S4a the electrochemical impedance spectra (EIS) of the CSC-S cell at OCV and after discharging to 1.0 V were compared. Further, the EIS of CSC-S (ultramicroporous) cell is compared with the CMK3-S (mesoporous) cell in order to see the effect of pore size on cell resistance (Fig. S4b). An additional bulk resistance is seen in the case of CSC-S cell discharged to 1.0 V, which could arise due to the reduced contact between the sulphur and electrolyte. In the case of ultramicroporous carbon (CSC-S) electrolyte could not diffuse into pores due to size constrain. Consequently the discharge and charge processes are much dependent on the bulk response. This could be the reason for the observed voltage hysteresis. Further, the notable decrease in the discharge and charge potentials of micropore confined sulphur in comparison to the cyclo-S_8_ reflects the change in thermodynamics due to the state of sulphur inside the micropores.

The initial discharge and charge capacities of the CSC-S composite were 1895 mAh g^−1^ and 1141 mAh g^−1^ respectively. The large irreversible capacity loss observed in the first cycle is likely due to the trapping of insulating Li_2_S_2_/Li_2_S well inside the narrow carbon pores. After the initial cycle the CSC-S composite shows a reversible discharge capacity of 1181 mAh g^−1^ at a rate of C/20. It is interesting to note that the first discharge capacity of microporous carbon based sulphur composites^41^,^48^,^49^,^50^ is consistently close to the theoretical specific capacity (1675 mAh g^−1^), whereas only 70–80% of the theoretical capacity in the initial discharge was observed for graphene^51^ or other mesoporous carbon^4^,^26^,^52^ sulphur composites. In our system, the first discharge capacity was 1895 mAh g^−1^. It is 220 mAh g^−1^ higher than the theoretical capacity of sulphur (1675 mAh g^−1^). The lithium insertion into the CSC host shows a discharge capacity of 90 mAh g^−1^ when discharged up to 1.0 V (Fig. S3). From this a calculated 65 mAh g^−1^ of the excess capacity for CSC-S cell is due to the lithium insertion into carbon. In order to examine the cause for the observed excess capacity the XPS analysis were performed on the electrodes after first discharge (unsputtered and sputtered) and after first cycle and compared with that of as-prepared electrode. Fig. S5 compares the XP spectra in the C 1s and O 1s region. The C 1s XP spectrum of the as-prepared electrode exhibited three components at binding energies of 287.0 eV, 289.0 eV and 291.0 eV corresponding to C–O, C = O and C–F, respectively. During discharge the peak at 289.0 eV corresponding to C = O disappeared due to the reaction with lithium. The peak at 287.0 eV remains even after discharge and the intensity slowly decreased with sputtering time. The peak at 291.0 eV corresponding to the C–F bond is due to the binder which disappeared on sputtering. Fig. S6 compares the XP spectra in the F 1s and P 2p region for as-prepared electrode, discharged to 1.0 V and recharged electrodes. The as-prepared electrode exhibited a single component in the F 1s XP spectrum due to the presence of binder (PVDF). The F 1s XP spectrum of the discharged electrode exhibited an additional component at 685.5 eV which was previously attributed to LiF^53^. After 2 min of sputtering time, surprisingly the intensity of LiF peak increased explicitly and remained constant with sputtering time. The peak corresponding to the binder disappeared slowly with sputtering time, which is consistent with the disappearance of C 1s peak at 291.0 eV with sputtering. The presence of LiPF_6_ is limited to the surface and its existence in the bulk is ruled out due to the disappearance of phosphorous peak with sputtering. The presence of LiF in the bulk of the materials could be related to the excess capacity observed during the first discharge. Further, LiF was found in the XP spectra of subsequently charged electrode confirming the irreversible formation of LiF in the first discharge. However, it is difficult to estimate the capacity contribution as the mechanism of formation of LiF is unclear at the moment.

Figure 3c shows the cycling behaviour of CSC-S composite obtained at C/5 rate. The first and second cycle discharge capacities are 1458 mAh g^−1^ and 927 mAh g^−1^, respectively, and it retained a reversible capacity of 703 mAh g^−1^ (75% capacity retention compared to the 2^nd^ cycle) after 100 cycles. A reversible capacity of about 411 mAh g^−1^ is still retained after 400 cycles with 44% of capacity retention compared to the 2^nd^ cycle. The average Coulombic efficiency for the initial 100 cycles was 99.4% and it remained constant at 99.6% in the subsequent cycles, indicating a superior cycling stability of the micropore confined sulphur compared to cyclo-S_8_. The observed stable (>99.4%) Coulombic efficiency of the CSC-S cathode at C/5 (Fig. 3b) indicates a minimal contact of carbonate based electrolyte with the micropore retained sulphur and the elimination of higher order polysulphide formation. This was further confirmed by examining the separator used in cells cycled to 10 and 400 cycles at C/5 rate. The photographs of the separator shown in Fig. 3e,f are not discoloured. The white colour of the separator was retained without any brown (typical colour of higher order polysulphides) tinge, even after 400 cycles, which indirectly proves the absence of any dissolution and shuttling effect of polysulphides. Further, the XPS analysis of the separator from the cycled cell (after 25 cycles) was performed to examine the presence of any soluble polysulphide (Fig. S7). The S 2p XP spectrum of the separator, contained two small peaks at binding energies of 163.8 eV and 169.5 eV corresponding to neutral sulphur and sulphate, respectively, and showed no peaks due to sulphide. The sulphur in the cycled separator could be due to the electrode particles stuck to the separator. No evidence for the existence of higher order polysulphide was observed.

The rate capability behavior of CSC-S composite at different current densities is shown in Fig. 3d. At low current densities of 83 and 166 mA g^−1^ the CSC-S composite delivered discharge capacities of 1200 and 1000 mAh g^−1^, respectively, which are comparable to other reported values^4^,^41^,^25^. By further increasing the rate to 1 C, a discharge capacity of 550 mAh g^−1^ was obtained. This is less compared to composites with graphene^51^/graphene oxide^54^,^55^ and CNT networks^56^ and might be due to the reduced electrical conductivity of the carbon host compared to the graphene based carbon backbone and due to the large pore length (particle size). It is important to note that no additional carbon was added during electrode fabrication. The rate capability could be further improved by increasing the conductivity of the carbon backbone and by reducing the pore length (particle size), the former could be achieved by altering the synthesis conditions that is by graphitizing the carbon at high temperature. Such studies are underway.

**Validation of lithiation and delithiation mechanism using XPS sputter profiling**

In order to understand the discharge and charge reaction mechanism of Li-S battery with micropore confined sulphur as cathode (and carbonate-based electrolyte), *ex-situ* XPS measurements were performed at various discharge and charge states during the first cycle. XPS analysis of cycled electrodes is often hampered by the interference of surface impurities and electrolyte decomposition products on the surface. Moreover, the bulk nature of the electrode and in particular the state of sulphur inside the sub-nanometer pores and its reactivity towards lithium have not yet been studied. Hence, in an attempt to probe the subsurface of the CSC-S composite, sputter profiling coupled with XPS analysis was carried out for the first time. This study gives new insight into the reaction and fading mechanism of the micropore confined sulphur cathode in the Li-S battery.

Figure 4 shows a series of XP spectra for the S 2p core level of electrode surfaces from cells discharged and charged to various states during the first cycle (as illustrated in Fig. 4a). The electrode surfaces were etched by Ar^+^ ion sputtering for 30  minutes to expose the fresh subsurface from which these XPS spectra were acquired. For reference, XPS measurements of pristine sulphur, CSC-S composite, pristine Li_2_S and Na_2_S_2_ were acquired (Fig. S8). The XP spectrum of elemental sulphur (Fig. S8a) exhibits two signals at 163.9 eV and 165.1 eV, which can be attributed to the S 2p_3/2_ and S 2p_1/2_ peak components, respectively. These characteristic peaks of neutral sulphur were also observed in the XP spectrum of the as-prepared CSC-S composite (Fig. S8b), which is in accordance to a previous report^57^. In addition, there was a broad peak with low intensity at ~168.5 eV due to the presence of some oxidized form of sulphur (S-O) on the surface of the unsputtered sample. The intensity of the peak due to S-O was significantly reduced after 3 minutes of sputter etching (Fig. S9). Furthermore, in the case of the sputtered as-prepared CSC-S composite (Fig. S9b,c) the presence of an additional small peak at lower binding energy (162.1 eV) could be due to the interaction of sulphur with the carbon host. The XP spectrum of pristine Li_2_S powder (Fig. S8c), exhibited two peaks couples with the S 2p_3/2_ peaks at binding energies of 160.7 ± 0.2 eV and 167.4 eV, corresponding to the sulphide (S^2−^) and oxy-sulphur (S-O) contamination, respectively. The S 2p core level peaks of Li_2_S are in agreement with the literature data^58^.

The S 2p XP spectrum of the as-prepared electrode (Fig. 4, position A), measured after 30 minutes of etching time, contained two components corresponding to neutral sulphur (S 2p_3/2_ at 163.9 eV, S 2p_1/2_ at 165.1 eV, major contribution) and S-C interaction (S 2p_3/2_ 162.1 eV, S 2p_1/2_ at 163.3 eV), which is in accordance to the as-prepared CSC-S composite (see Fig. S9). Also the spectra before sputtering were in complete agreement with the results of the composite before electrode preparation (Fig. S10). The XP peak at 162.1 eV corresponding to the sulphur carbon interaction in the CSC-S composite is corroborated by the EELS spectra (Fig. 2d) with an increase in the σ* peak intensity.

Furthermore, the cells were discharged to various voltages (see Fig. 4a) and XP spectra were acquired. At positions B and C, the S 2p XP spectra were deconvoluted into two components associated to neutral sulphur and a polysulphide (S 2p_3/2_ at 162.2 eV). At discharge positions D and E, the XP spectra in the binding energy region of the S 2p core levels revealed three components which were assigned to neutral sulphur, a polysulphide (S 2p_3/2_ at 162.2 eV) and Li_2_S (S 2p_3/2_ 160.9 eV). During discharge, the electrodes analyzed along the length of the plateau (position B, C and D) exhibited only a single intermediate polysulphide that directly converted to Li_2_S at the end of the discharge. The binding energy of polysulphide (S 2p_3/2_ at 162.2 eV) formed during the discharge is close to the binding energy of Na_2_S_2_ (S 2p_3/2_ at 162.0 eV) (Fig. S8d) and hence the composition of the polysulphide is confirmed as Li_2_S_2_. Near the end of discharge the formation of Li_2_S is confirmed by comparing its binding energy with commercial Li_2_S (Fig. S8c). No indications were found for the formation of higher order polysulphides during discharge either on the surface, subsurface of the electrode or in the separator. It is important to note that during discharge the Li_2_S_2_ peak is growing at the same binding energy (S 2p_3/2_ 162.1 eV, attributed to carbon-sulphur interaction) of the sputtered as-prepared electrode (Fig. 4, position A) which is further converted to Li_2_S at the end of the discharge. From the XPS results and from the discharge sequence we postulate that the interaction between sulphur and carbon assists in the nucleation and further direct formation of Li_2_S_2_/Li_2_S. Further, in the absence of direct contact between sulphur and liquid electrolyte, the reaction between sulphur and lithium is quasi solid-state and direct transformation of sulphur to lower order polysulphide is possible.

The electrode of the cell discharged and charged to position F exhibited two components in the S 2p XP spectrum, associated to a polysulphide (Li_2_S_2_, S 2p_3/2_ 162.2 eV) and neutral sulphur. At this stage, the peak couple due to Li_2_S disappeared corresponding to the complete oxidation of Li_2_S. At fully charged state (position G), peaks corresponding to polysulphides (S 2p_3/2_ 162.2 eV) and neutral sulphur appeared, but with a substantial loss in intensity in the polysulphide related S 2p_3/2_ peak. The presence of unreacted Li_2_S_2_ even after complete charge could be a reason for the observed irreversible capacity loss in the first cycle.

**Fading mechanism**

The capacity of the Li-S battery with CSC-S cathode decreased during cycling (Fig. 3c) despite the elimination of higher order polysulphide. In order to elucidate the cause for capacity fading the cathodes of the cells after 10 and 400 discharge/charge cycles were examined using XPS. The S 2p XP spectra of the cycled electrodes measured before (a, b) and after sputtering (c, d) for 30 minutes are presented in Fig. 5. Before sputtering the surface of the electrode after 10 discharge/charge cycles (Fig. 5a) exhibited two doublet peaks, with the S 2p_3/2_ peaks at binding energies of 163.9 eV and ~170 eV corresponding to neutral sulphur and sulphate, respectively. The spectra measured after etching (Fig. 5c) exhibited a single peak that contained two components corresponding to the neutral sulphur and the polysulphide (Li_2_S_2_, 162.2 eV). The peak due to sulphate, which might have formed during sample loading, disappeared in the sputtered sample (Fig. 5c). From XPS it is clearly evident that the discharged products and sulphur are confined inside the carbon micropores. The presence of such species is not observed in the XP spectra measured without sputtering.

The S 2p XP spectrum (Fig. 5b) of the electrode surface after 400 discharge/charge cycles (charged state) exhibited a single peak containing two components, which correspond to neutral sulphur and a polysulphide (Li_2_S_2_, 162.2 eV). After 30 minutes of sputtering, an additional peak grew in, corresponding to Li_2_S (160.9 eV). Since the subsurface of the electrode is probed after sputter etching, this result demonstrates the presence of a discharge product and sulphur which are trapped inside the carbon micropores. When comparing the results after 10 and 400 discharge/charge cycles, it is evident that the discharged product and sulphur accumulated over charge and discharge, respectively during cycling and thereby contributed to the observed capacity fading. To further assess the reason for the observed capacity fading EIS of the CSC-C cells were monitored with cycling. Fig. S4c compares the EIS of cells measured after 1^st^, 176 and 400 cycles. The resistance is reducing gradually with cycling. Similar observations were made by Li *et al.*, in the case of microporous carbon-confined sulphur composites during cycling^49^. This could be related to the accumulation of relatively low resistant products with cycling. In addition, the lowered access of the electrolyte to the active material (sulphur and discharge products) confined into the long narrow micropores of carbon could also contribute towards capacity fading. The later contribution could be minimized by choosing small particle size carbon host with improved internal electronic conductivity.

The confinement of the discharge product in the micropores of carbon was also indirectly supported by the absence of any morphological/surface change due to the accumulation of Li_2_S on the electrode surface. Fig. S11 shows SEM micrographs of the as-prepared CSC-S composite and of the surface of electrodes after 35 and 400 cycles. The morphology remained similar in all three cases with no segregation of crystalline Li_2_S on the surface of the cycled electrodes.

## Conclusions

We have examined and validated the lithiation/delithiation mechanism and the mechanism of capacity fade over cycling for sulphur confined in ultramicroporous carbon cathode using XPS sputter profiling. The single step transformation of sulphur to Li_2_S_2_/Li_2_S and vice-versa during the discharge/charge process is explained by analyzing the subsurface using XPS. In addition, we postulate that the interaction between sulphur and carbon assists in the nucleation and further direct formation of Li_2_S_2_/Li_2_S. Further, in the absence of direct contact between sulphur and liquid electrolyte, the reaction between sulphur and lithium is quasi solid-state and a direct transformation of sulphur to lower order polysulphide is possible. The discharged product and sulphur accumulated inside the narrow micropores are a major cause for capacity fading in microporous carbon-sulphur composites. The results presented here can help to improve the design of carbon templates for sustainable sulphur cathode in Li-S battery. High capacity and high Coulombic efficiency could be achieved by circumventing the formation of higher order polysulphides and by reducing the particle size with improved electronic conductivity thereby mitigating the trapped insulating intermediates in carbon pores, respectively.

## Methods

### Synthesis of activated ultra-microporous carbon (CSC)

Coconut shells were used as raw material to produce ultra-microporous carbon. Water washed and dried fibre-free coconut shells were crushed to lumps with a size less than 5 mm. The crushed pieces were further powdered by ball-milling at 300 rpm for 12 h. The obtained powder was carbonized at 600 °C for 2 h under Ar flow at a heating rate of 5 °C min^−1^. The carbonized material was activated by employing KOH as chemical activating agent which is established to generate microporous carbon^42^. For this purpose, the resulting carbonized material was mixed with KOH at a weight ratio of 1:4 and heated to 400 °C for 1 h and at 800 °C for 2 h under an Ar flow at a heating rate of 5 °C min^−1^. After cooling to room temperature, the product was neutralized using diluted HCl and washed repeatedly with distilled water, centrifuged and dried at 100 °C.

### Synthesis of CSC-S composite

The CSC-S composite was prepared by vacuum assisted melt infusion method. The as-prepared carbon and sulphur were ground together in the mass ratio of 1:1 and transferred to a rotating oven (Buchi, B-585 Kugelrohr). The mixture was evacuated to 10^−3^ mbar. The temperature was raised to 155 ± 1 °C from RT, held at this temperature for 12 h and cooled back to RT after that. The long heating time and low pressure maintained during the composite synthesis facilitates the infusion of sulphur melt into the carbon micropores^25^,^40^,^41^.

### Characterization

The X-ray diffraction (XRD) patterns were obtained in transmission mode using Cu K_α_ (λ = 0.15406 nm) radiation on a STOE STADI P diffractometer. Nitrogen sorption isotherms were obtained using a Micromeritics ASAP 2020 analyser. Specific surface areas were calculated using the Brunauer- Emmett-Teller (BET) method, and the pore size distributions were calculated by the density functional theory (DFT) method. The morphology of carbon and carbon sulphur composite were examined by scanning electron microscopy (SEM, LEO 1550 Gemini). Transmission electron microscopy (TEM) and electron energy-loss spectroscopy (EELS) were carried out using an image side Cs corrected FEI-TITAN 80–300 equipped with a GIF Quantum 965 energy filter and operated at 80 kV to avoid knock-on damage and increase the energy resolution. Thermogravimetric analysis and differential scanning calorimetry (TGA-DSC) measurements for the composite were carried out using a Sensys Evo TG-DSC apparatus (Setaram, France). The elemental composition of the sample surfaces was determined by X-ray Photoelectron Spectroscopy (XPS) measurements using monochromatized Al K_α_ (1486.6 eV) radiation (PHI 5800 MultiTechnique ESCA System, Physical Electronics). The measurements were done with a detection angle of 45°, using pass energies at the analyzer of 93.9 and 29.35 eV for survey and detail spectra, respectively. For binding energy calibration the C(1s) peak was set to 284.8 eV. The sample surfaces were sputtered for 30 minutes (5 kV, 1 μA) for subsurface analysis.

### Electrochemical measurements

Electrochemical studies were performed in Swagelok type cells. Electrode fabrication and assembly of electrochemical cells was done in an argon filled glove box. The positive electrodes were fabricated by mixing the CSC-S composite (45.8% of S) with the binder, poly vinylidene fluoride (PVDF) in a weight ratio of 90:10. A slurry containing the above mixture was prepared by using N-methyl-2-pyrrolidinone as solvent, spread on a stainless steel (SS) foil (area: 1.13 cm^2^), and dried in an oven at 90 °C for 12 h. Typically, each electrode contained 1.5–2.0 mg of sulphur. The specific capacities were calculated on the basis of the sulphur mass in the electrode. A lithium foil (Aldrich, 99.9%) was used as the negative electrode, and a borosilicate glass fiber sheet saturated with 1 M LiPF_6_ in 1:1 ethylene carbonate (EC)/dimethyl carbonate (DMC) (LP30, BASF) was used as separator and electrolyte. The cells were placed in an incubator (Binder) to maintain a constant operation temperature of 25 ± 0.1 °C. The cells were discharged and charged between 1.0 and 3.5 V vs Li^+^/Li at different cycling currents using an Arbin battery cycling unit BT2000.

## Additional Information

**How to cite this article**: Helen, M. *et al.* Single step transformation of sulphur to Li_2_S_2_/Li_2_S in Li-S batteries. *Sci. Rep.* **5**, 12146; doi: 10.1038/srep12146 (2015).

## Supplementary Material

Supplementary Information

## Figures and Tables

**Figure 1:**
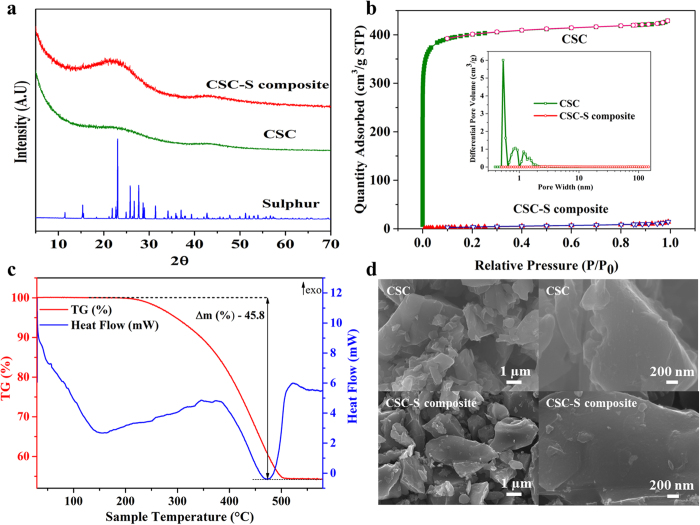
XRD pattern, adsorption isotherm, thermogravimetric analysis and morphology of CSC-S composite. **a**) XRD patterns of pure sulphur, CSC and CSC-S composite. (**b**) N_2_ adsorption/desorption isotherm for CSC and CSC-S composite. Inset: Pore size distribution. (**c**) Thermogravimetric analysis (TGA) of CSC-S composite performed under Helium flow of 20 ml min^−1^ with a heating rate of 10 °C min^−1^. (**d**) SEM images of CSC and CSC-S composite.

**Figure 2:**
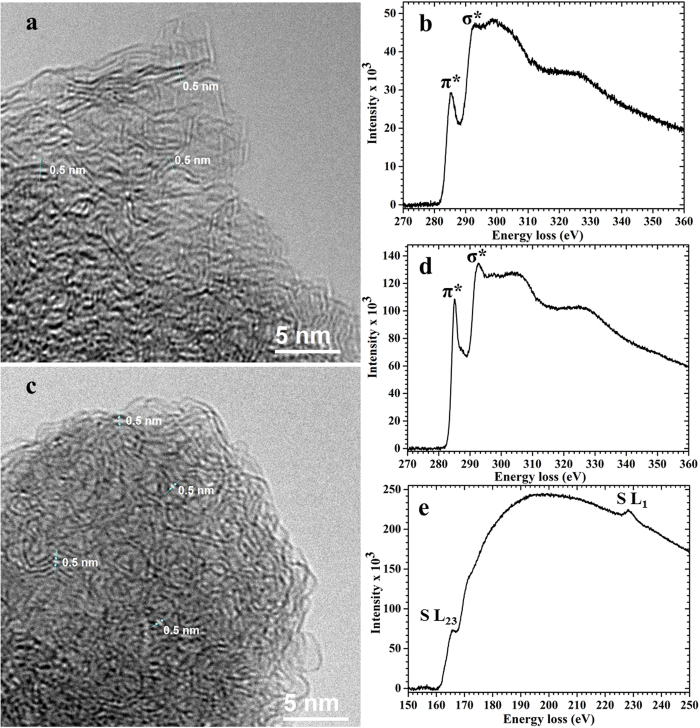
TEM micrographs and power-law background subtracted EELS spectra of the as-synthesized CSC and the CSC-S composite. (**a**) As-synthesized CSC. (**b**) C-K edge of as-synthesized CSC. (**c**) CSC-S composite. (**d**) C-K edge of CSC-S composite and (**e**) S-L_1,2,3_ edge of CSC-S composite.

**Figure 3:**
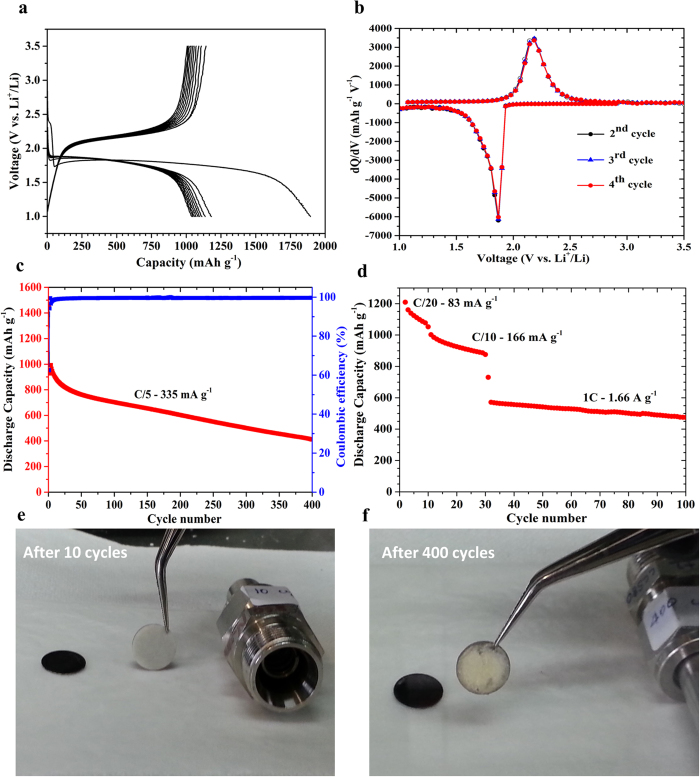
Electrochemical performance of a CSC-S composite cathode using 1.0 M LiPF_6_ in EC/DMC (1:1 v/v) as electrolyte. (**a**) Discharge/charge profiles of CSC-S composite at C/20 (84 mA g^−1^). (**b**) Differential capacity plots (DCP) for the 2^nd^, 3^rd^ and 4^th^ discharge/charge cycle. (**c**) Cycling performance and Coulombic efficiency at C/5 (335 mA g^−1^). (**d**) Rate capability of the cell at various discharge rates. The voltage range is 1 to 3.5 V vs Li^+^/Li. The capacity values are given related to the sulphur mass. (**e**,**f**) Pictures of the separators after 10 and 400 cycles.

**Figure 4:**
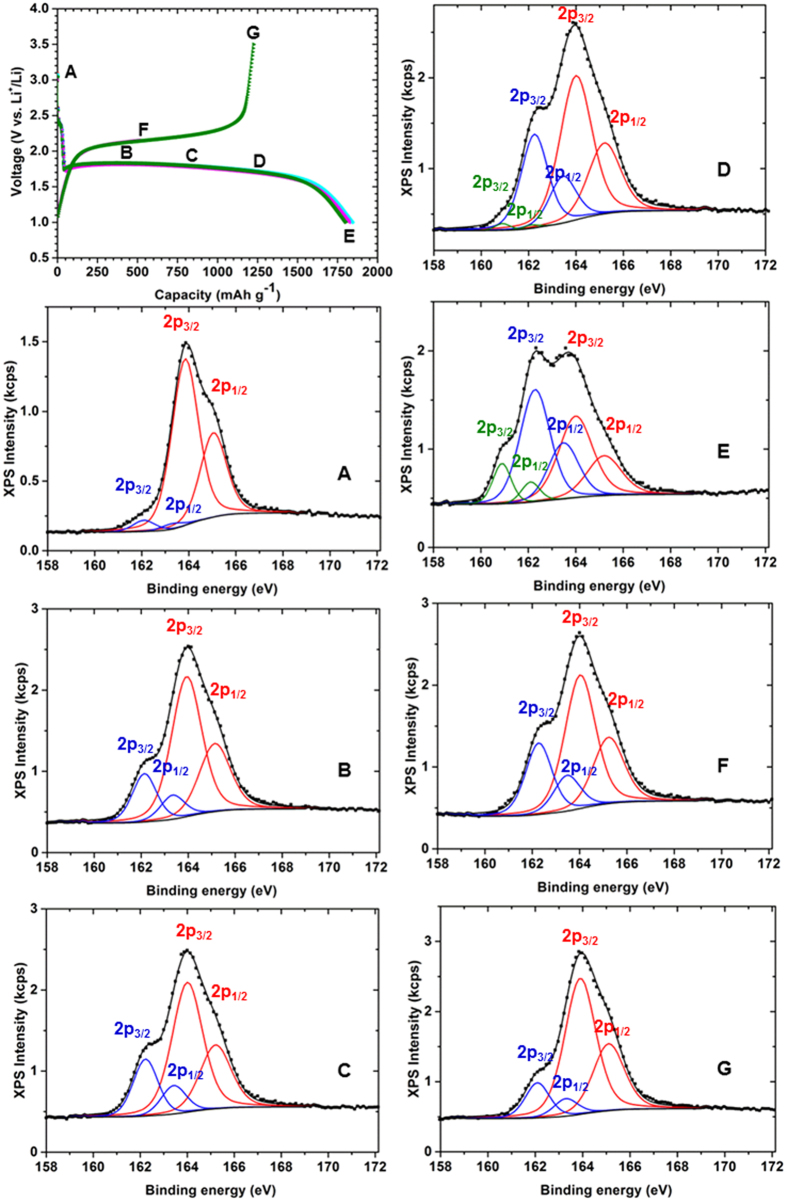
*Ex-situ* XPS measurements of CSC-S composite cathode during the first discharge and charge cycle. A voltage profiles of cells discharged and charged to various states (at C/20) are indicated. (**A–G**), XP spectra of the S 2p core-level of the cathode at various discharged and charged states (as denoted in a).

**Figure 5:**
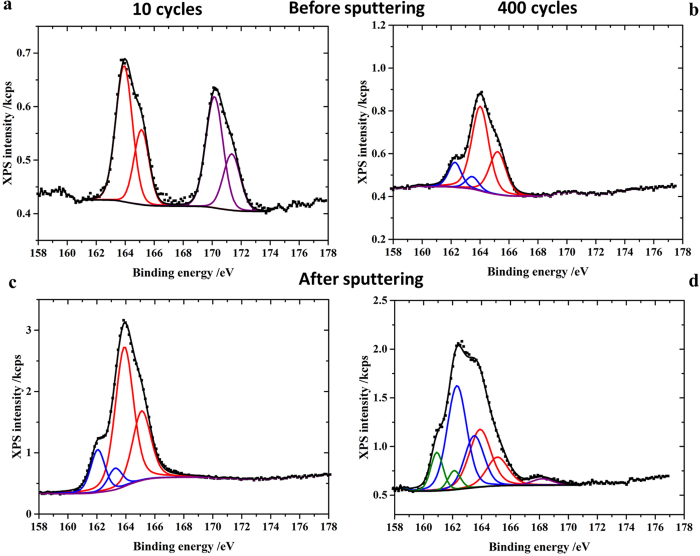
*Ex-situ* XPS measurements of cathodes after 10 and 400 discharge/charge cycles. **a**,**b**) XP spectra of the S 2p core-level of the cycled cathode before sputtering and (**c**,**d**) after 30 minutes of sputtering.
