# Supplementary material for: Single step transformation of sulphur to Li2S2/Li2S in Li-S batteries
Source: Sci Rep. 2015 Jul 15;5:12146. doi: 10.1038/srep12146 (PMC4502410; doi:10.1038/srep12146)

Single step transformation of sulphur to Li_2S_2/Li_2S in Li-S batteries

M. Helen[†]*, M. Anji Reddy[†], Thomas Diemant[§], Ute Golla-Schindler[⊥], R. Jürgen Behm[†§], Ute Kaiser[⊥], and Maximilian Fichtner[, †‡]*

[†] *Helmholtz Institute Ulm (HIU), D-89081 Ulm, Germany*
[§] *Institute of Surface Chemistry and Catalysis, Ulm University, D-89081 Ulm, Germany*
[⊥] *Electron Microscopy Group of Materials Science, Central Facility for Electron Microscopy, Ulm University, D-89081 Ulm, Germany*
[‡] *Institute of Nanotechnology, Karlsruhe Institute of Technology, P.O. Box 3640, D-76021 Karlsruhe, Germany*

** Corresponding author. Email address: helen.joseph@kit.edu, Tel.: +49 (0)731 50 34215*
Email address: maximilian.fichtner@kit.edu, Tel.: +49 (0)731 50 34201

Figure S1. TEM micrographs of the as-synthesized CSC and the CSC-S composite.

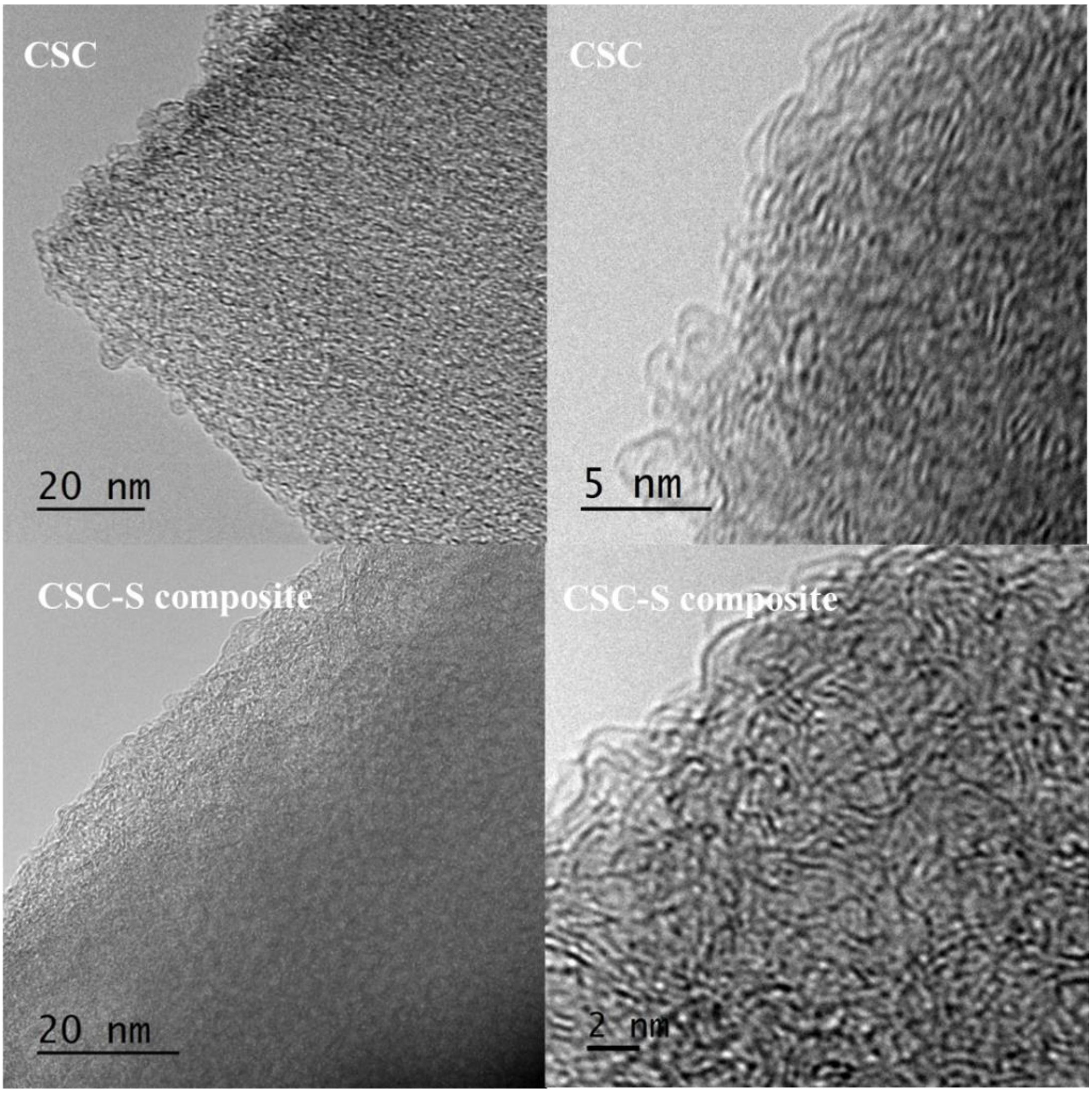

Figure S2. Spliced raw data EELS of (a) as-synthesized CSC and (b) CSC-S composite, the pronounced S peak in the CSC-S sample is clearly seen.

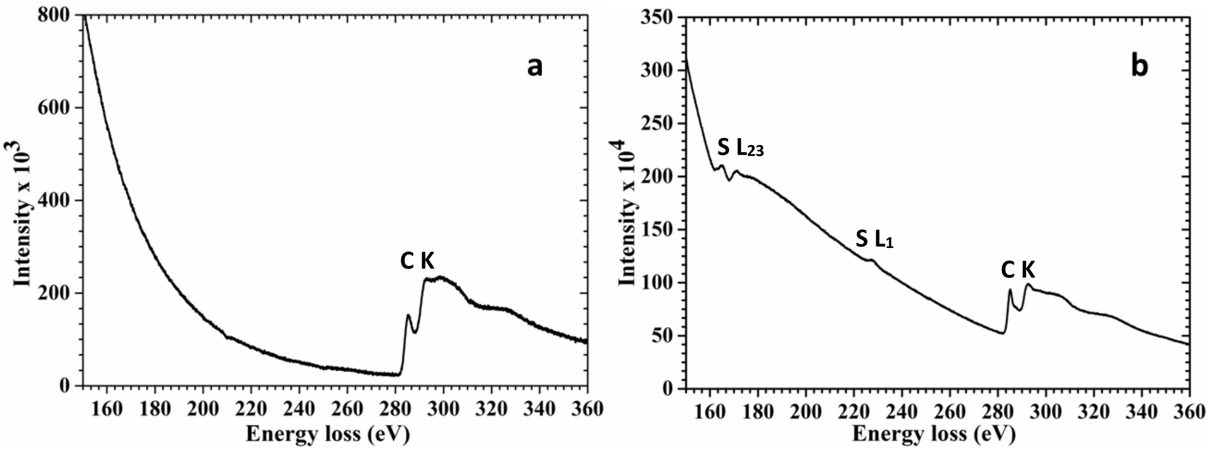

Figure S3. First lithium insertion into the as-synthesized ultramicroporous carbon (CSC) host.

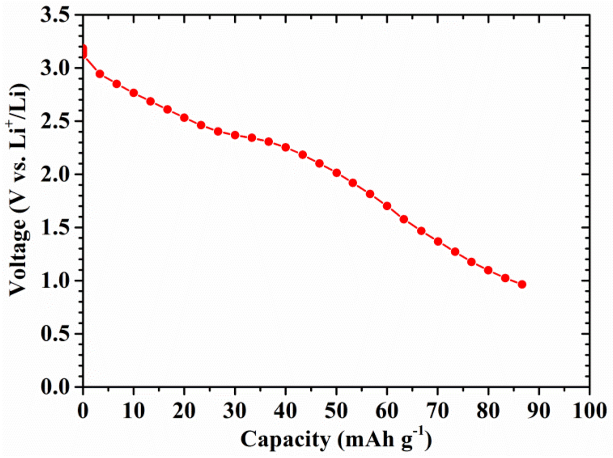

Figure S4. Electrochemical impedance spectra of cells (a) with CSC-S at OCV and after discharged to 1.0 V, (b) with CSC-S and CMK3-S discharged to 1.0 V and (c) with CSC-S at various cycles.

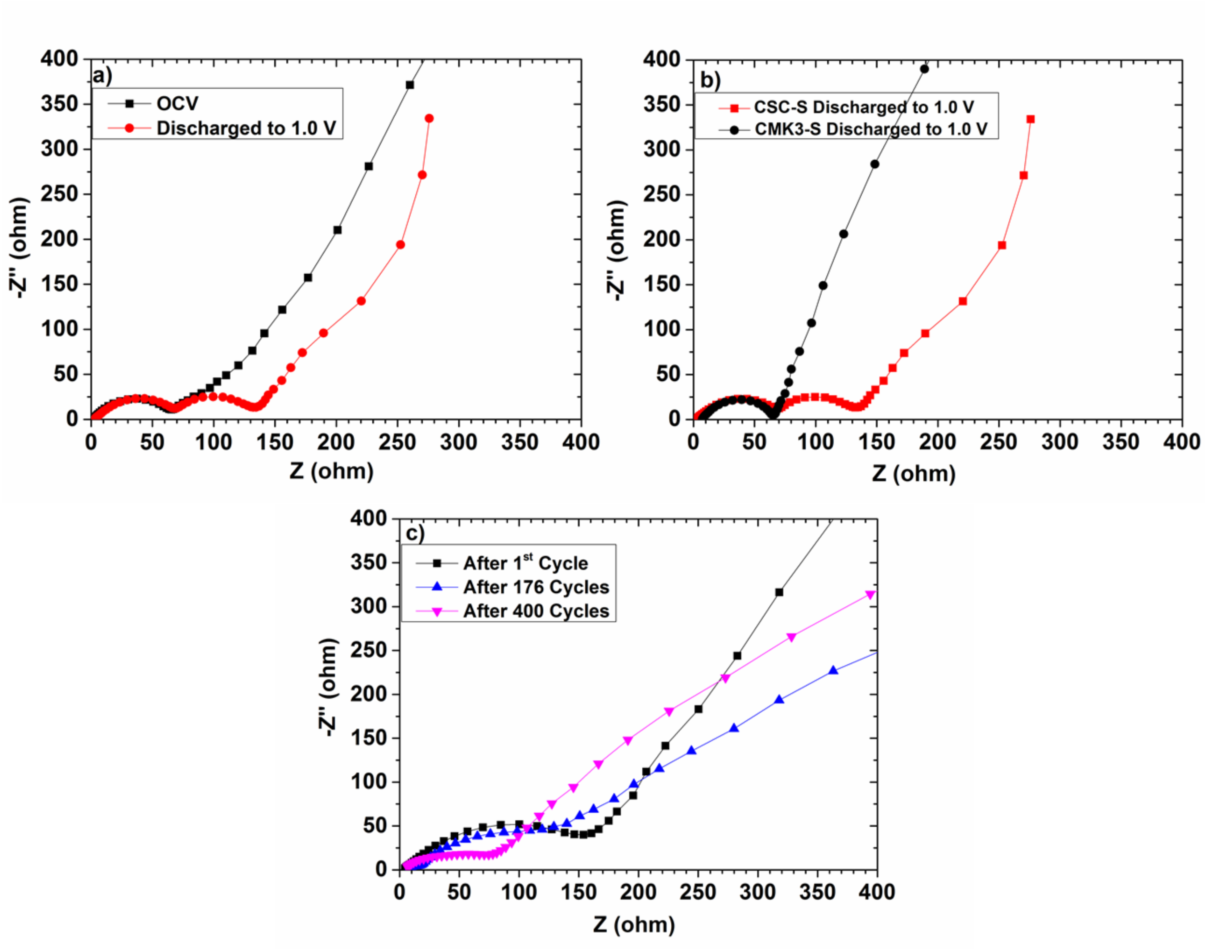

Figure S5. Comparison of XP spectra in the C 1s and O 1s region for as-prepared electrode, discharged to 1.0 V and recharged electrodes.

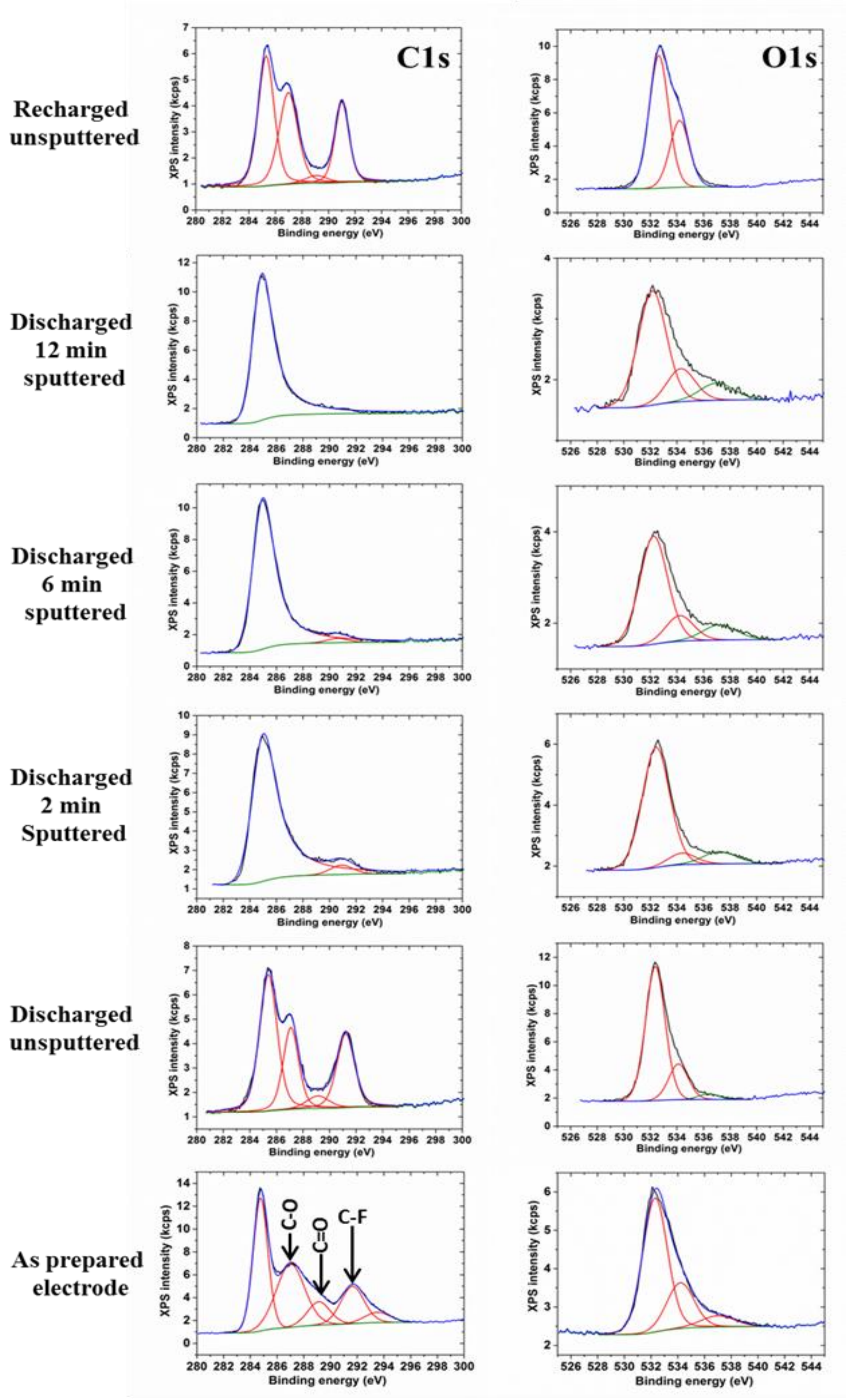

Figure S6. Comparison of XP spectra in the F 1s and P 2P region for as-prepared electrode, discharged to 1.0 V and recharged electrodes.

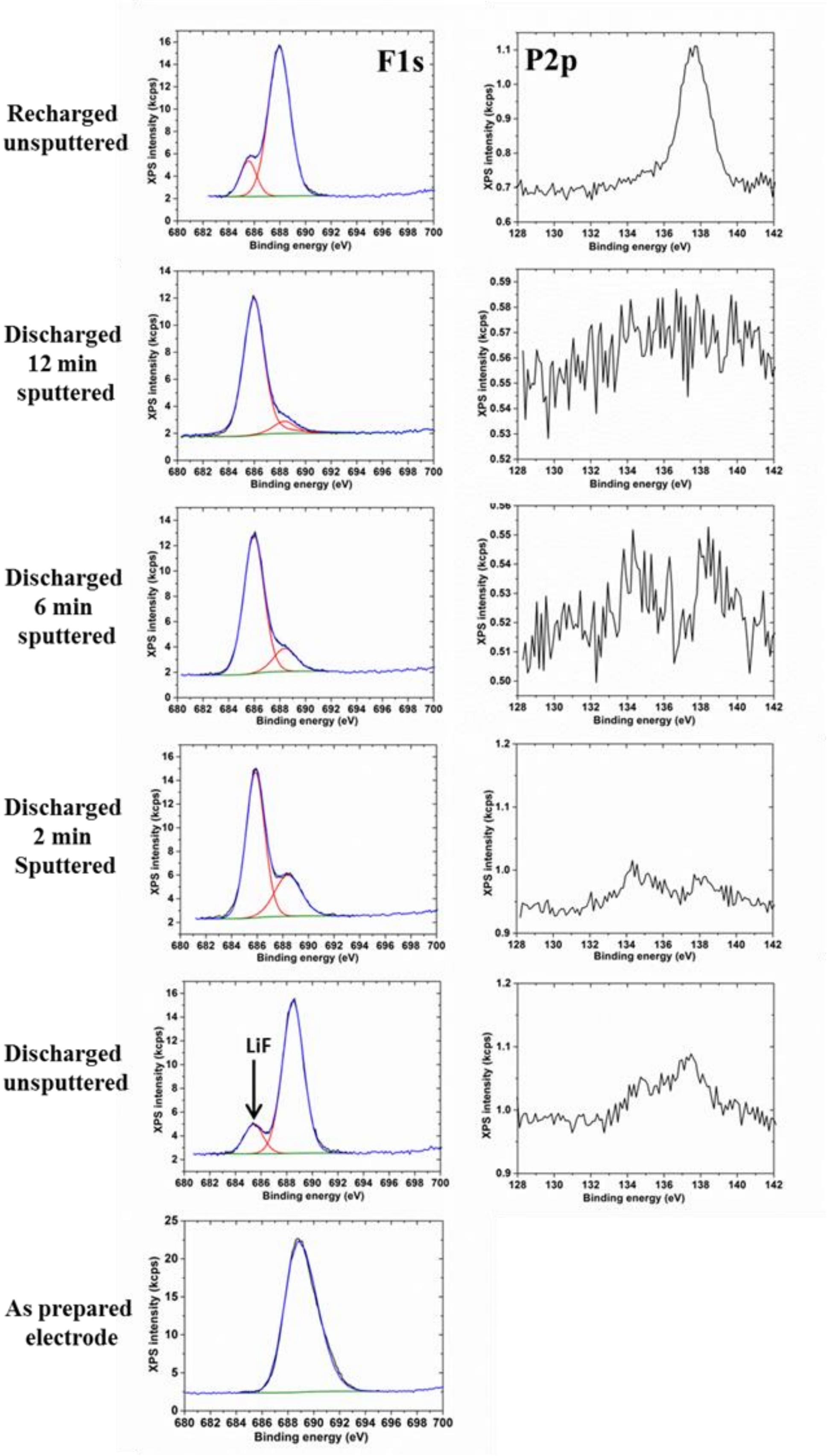

Figure S7. XP spectra in the S 2p region recorded for the separator from the CSC-S cell after 25 cycles.

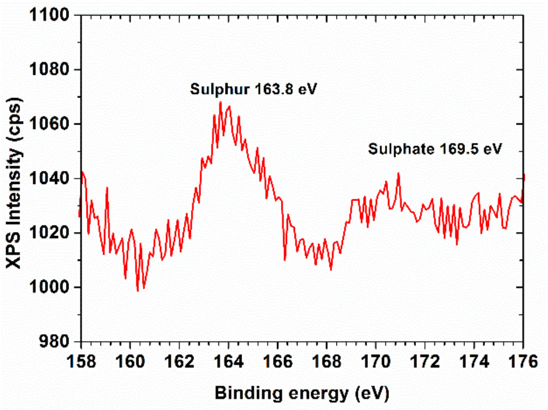

Figure S8. XP spectra in the S 2p region recorded for (a) pristine sulphur, (b) CSC-S composite, (c) pristine Li_2S and (d) Na_2S_2. The S $2p_{3/2}$ peaks at binding energies of 163.9 eV and ~ 168 eV corresponds to neutral sulphur and sulphate, respectively. The XP spectra in the S 2p region for Na_2S_2 represent the S_2^{2-} at binding energy 162.0 eV along with the S^{2-} in Na_2S (impurity) and elemental sulphur (reactant used in Na_2S_2 synthesis) exhibiting S $2p_{3/2}$ binding energies at 160.0 and 164.0 eV, respectively.

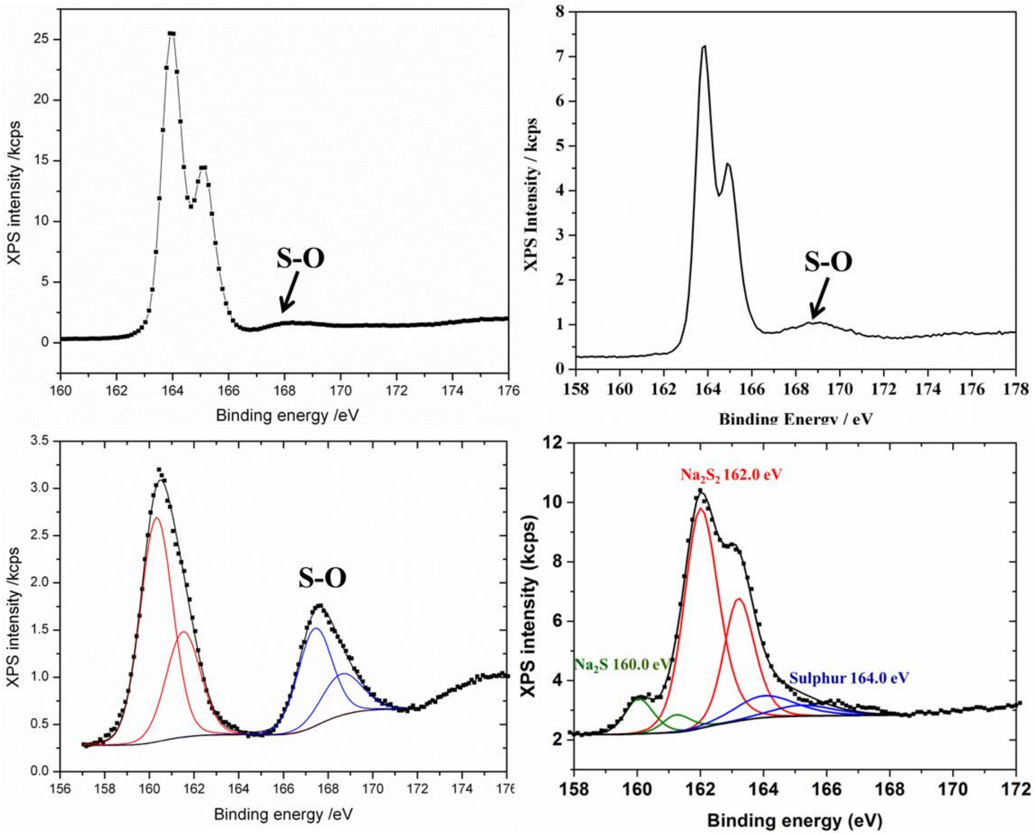

Figure S9. XP spectra in the S 2p region recorded for CSC-S composite before sputtering and after 3 & 10 min of sputtering. After sputtering the S-O peak on the surface disappeared and the peak corresponding to S-C appeared.

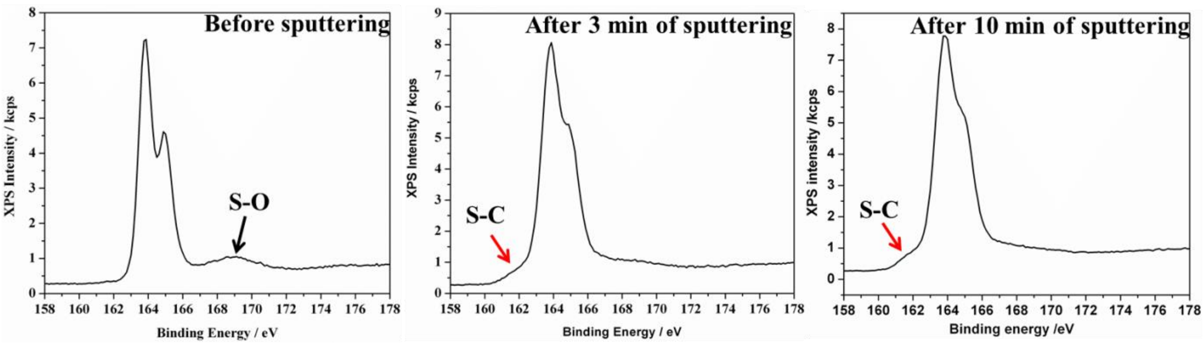

Figure S10. XP spectra in the S 2p region recorded for the as-prepared electrode before sputtering and after 30 min of sputtering

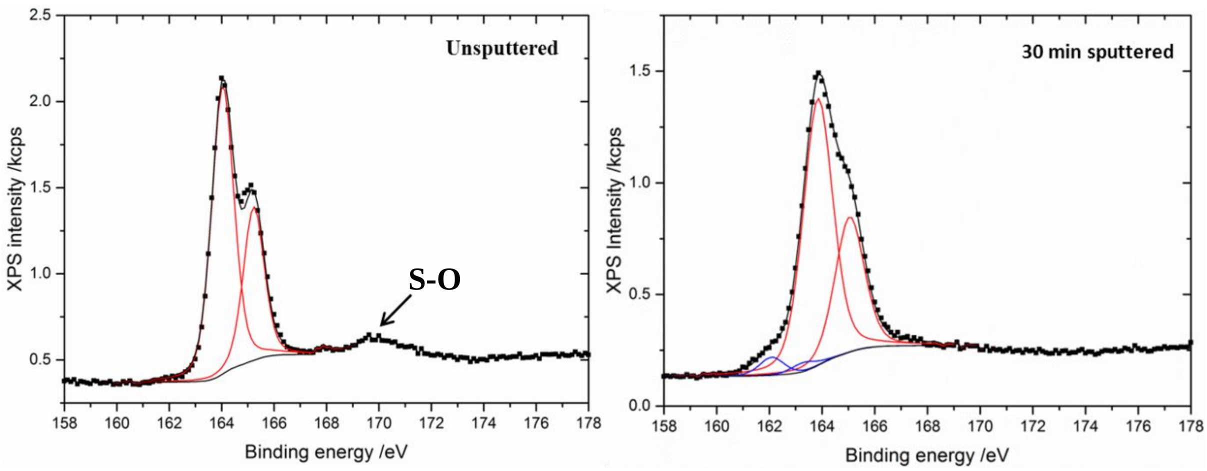

Figure S11. SEM micrographs of the as-prepared CSC-S composite in comparison to electrodes discharged and charged to 35 and 400 cycles.

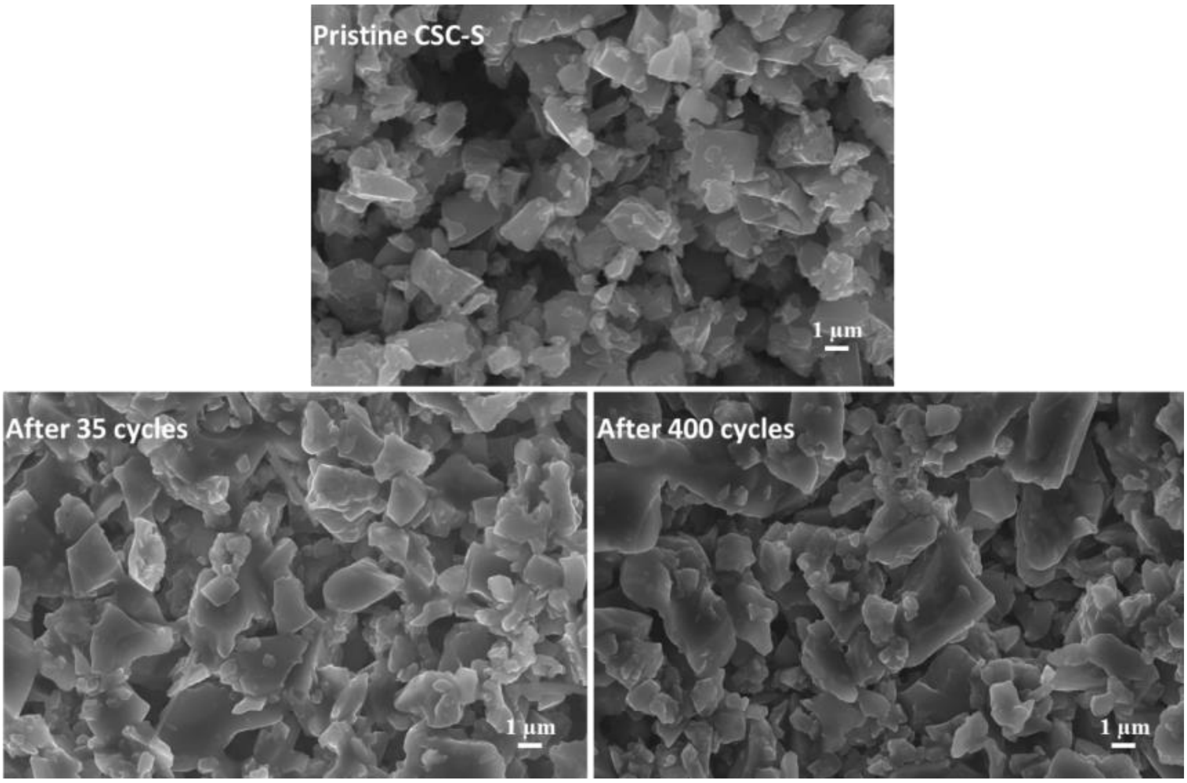

Supplement: Supplementary Information [file srep12146-s1.pdf]
